# Antimicrobial effects of chlorine dioxide in a hospital setting

**DOI:** 10.1038/s41598-023-49997-z

**Published:** 2023-12-18

**Authors:** Mariana Helou, Ahmad Mahdi, Antoine Abou Fayad, Ahmad Sleiman, Ghassan M. Matar, Sanaa Zoghbi, Tarek Madani, Rola Husni

**Affiliations:** 1 Division of Emergency, Department of Internal Medicine, School of Medicine, Lebanese American University, Beirut, Lebanon; 2 Division of Infectious Diseases, Department of Internal Medicine, School of Medicine, Lebanese American University, Beirut, Lebanon; 3 Center for Infectious Diseases Research, American University of Beirut, Beirut, Lebanon; 4 Infection Control Program, Lebanese American University Medical Center, Beirut, Lebanon; 5 Lebanese American University-Rizk Hospital, Beirut, Lebanon

**Keywords:** Health policy, Health occupations

## Abstract

Chlorine dioxide is a powerful disinfectant with strong antibacterial properties. We conducted a study at different sites of the Lebanese American University Medical Center-Rizk Hospital to determine the efficacy of the ECOM air mask in decreasing the particle load. Air cultures were obtained from three different locations, namely the patients’ elevator, visitors’ elevator and mobile clinic and the number of colonies grown on each type of agar was determined. We also measured particle counts at the three sites both at baseline and after placement of the ECOM air mask. After 7 days of ECOM air mask use, the numbers of colonies grown on all types of media was decreased by 20–100% versus the baseline values. The counts of particles of different diameters (0.3, 0.5 and 5 µm) were decreased at all three sampled sites. This study highlighted the efficacy of the ECOM air mask. The utility of the gaseous form of ClO_2_ as an antiseptic in the hospital setting appears promising.

## Introduction

Chlorine dioxide (ClO_2_) is a powerful disinfectant that disrupts protein synthesis and cell membranes and a strong antioxidant that inhibits microbial growth. ClO_2_ has demonstrated antiviral, antibacterial and antifungal properties in vitro. It is produced by an acid–base reaction or through the use of an electrolytic method^1^. ClO_2_ can be used in an aqueous or gaseous state. As a gas, it has a yellow to reddish-yellow colour at room temperature and it has proven sporicidal effects at concentrations of 10–40 mg/L and contact times of at least 30 min on metal, plastic and glass surfaces^2^. In one study, ClO_2_ exhibited strong antibacterial properties attributable to its protein-denaturing ability, which is important for disturbing spore viability and/or integrity^3^. In another study, gaseous ClO_2_ at an extremely low concentration prevented influenza a virus infection in mice caused by aerosolised particles^4^.

The maximum residual disinfectant level of ClO_2_ is 0.8 mg/L according to the disinfectants and disinfection by-products rule. The ClO_2_ permissible exposure limits defined by the Occupational Safety and Health Administration are 0.1 ppm and 0.3 mg/m^3^ for general industry^1^. Even with a low concentration of chlorine dioxide, it is proven effective against micro-organisms^5^.

The COVID19 pandemic is considered one of the most challenging healthcare crises of the twenty-first century. The rapid and global spread of the disease both jeopardised global health and imposed socioeconomic challenges^6^. In Lebanon, our ‘Emergency Risk Management Programme’ plan proved inadequate to tackle infectious disease pandemics and the healthcare system was already overloaded prior to the onset of the pandemic, as the country featured the highest ratio of refugees per capita globally. Therefore, the COVID19 pandemic had serious consequences on the healthcare system and the economy^7^. Chlorine dioxide was proven to be an effective disinfectant during the COVID-19 pandemic^5^.

Infection control practices should ensure high standard of hygiene at the hospital level for patients and staff safety. In addition, antimicrobial resistance (AMR) is a worldwide rising concern requiring mitigation measures at the level of infection control. According to the Infectious Diseases Society of America, AMR represents one of the biggest threats to human health, as patients infected with multidrug-resistant (MDR) organisms have worse prognoses. Typically, MDR bacteria are more common in the healthcare setting and all efforts should be exerted to prevent their spread to the community^8^. A study conducted at Wolaita Sodo University Teaching and Referral Hospital revealed that aerosol transmission can contribute to the persistence of bacterial pathogens in the hospital environment and the rise of various healthcare-associated infections. The study additionally identified a high prevalence of airborne bacteria of clinical concern, such as *Staphylococcus aureus*, *Enterococcus*, *Acinetobacter*, *Pseudomonas aeruginosa* and *Escherichia coli*, which contributed to a high prevalence of post-surgical site and respiratory tract infections in the study area^9^. MDR bacteria have been a major concern among patients with COVID-19.

Our study is novel. No previous studies were conducted about the gaseous ClO_2_ use in a hospital setting. The ES-010 ECOM air mask is a device that emits 0.017–0.031 ppm of ClO_2_.

Therefore, we examined the efficacy of the gaseous form of ClO_2_ in killing bacteria and fungi. The aim of this study was to determine the efficacy of the ECOM air mask in decreasing the load of particles in air and the number of bacterial and fungal colonies grown on different types of agar from air samples at the Lebanese American University Medical Center-Rizk Hospital.

## Materials and methods

### Effect of the ECOM air mask on microorganisms

To perform this experiment, MacConkey agar (MAC) was used to isolate gram-negative bacteria, trypticase soy agar (TSA) and Luria–Bertani agar (LB) were used to culture different types of microorganisms and Sabouraud agar (Sab) was used to grow pathogenic and non-pathogenic fungi. Samples were collected from the patients’ elevator, visitors’ elevator and mobile clinic, one sample from each location. Prior to placement of the ECOM air mask, the baseline level of microbes was recorded by taking air cultures using SPIN AIR V2 (absorbs 100 L of air in 1 min) on all four types of agar. SPIN AIR V2 is a portable device, that can retrieve accurate volumes of air by forcing them, and after incubation, colony enumeration is done. The ECOM air mask was placed in the middle of each sampling area and samples were taken again after 1 week. The agar plates were incubated at 37 °C for 18 h and the number of colonies that grew on each medium was counted.

### Effect of the ECOM air mask on particles

To detect the antibacterial and antifungal activity of 0.017–0.031 ppm ClO_2_ emitted from the ES-010 ECOM air mask, we measured the particle count at the three aforementioned locations (patient elevator, visitor elevator, mobile clinic). First, baseline samples (T0) were obtained, followed by placement of the ECOM air mask. In both elevators, samples were taken 2, 4, 5, 6, 8, 20 and 25 days after placing the ECOM air mask. For the mobile clinic, samples were taken 10, 20 and 24 h and 4 and 15 days after placing the ECOM air mask. The particles were counted using the TSI AeroTrak® 9303 Handheld Particle Counter, which measures all particles based on a set diameter. The TSI AeroTrak® 9303 Handheld Particle Counter is a lightweight plastic device used for particle measurements. The instrument can report up to three particle sizes simultaneously.

## Results

### Effect of the ECOM air mask on the microorganism count

As shown in Table 1, After 7 days of ECOM air mask use, the numbers of colonies grown on all types of agar decreased by 20–100% versus the initial values.

**Table 1:** Number of colonies grown from samples at different locations before and 7 days after placement of the ECOM air mask.

Location	Type of petri plate	Number of colonies		Percentage decrease in number of colonies
		Baseline	After 7 days of ECOM air mask	
Patients Elevator	Luria–Bertani agar (LB)	272	217	20%
	Trypticase soy agar (TSA)	319	88	72%
	Sabouraud agar (Sab)	189	1	99%
	Macconkey agar (MAC)	9	0	100%
Visitors Elevator	Luria–Bertani agar (LB)	212	139	34%
	Trypticase soy agar (TSA)	267	56	79%
	Sabouraud agar (Sab)	187	1	99%
	Macconkey agar (MAC)	7	0	100%
Mobile Clinic	Luria–Bertani agar (LB)	265	34	87%
	Trypticase soy agar (TSA)	162	37	77%
	Sabouraud agar (Sab)	2	11	
	Macconkey agar (MAC)	0	2	

### Effect of the ECOM air mask on particles in air

The counts of particles of three different diameters (0.3, 0.5 and 5 µm) were recorded at baseline and at several subsequent time points, as in Tables 2, 3, 4.

**Table 2:** Number of particles after placement of the ECOM air mask in the mobile clinic.

Mobile clinic	Time (h/days)					
Measurements (*10^3^ particles/m^3^)	T0	T10 h	T20 h	T24 h	T96 h	T15 days
Small 0.3 µm	170,284	166,042	156,763	137,551	184,990	217,557
Medium 0.5 µm	27,471	17,968	15,251	14,724	23,173	60,554
Large 5 µm	168	73	73	175	51	86

**Table 3:** Number of particles after placement of the ECOM air mask in the visitors’ elevator.

Visitors elevator	Time (days)							
Measurements (*10^3^ particles/m^3^)	T0	2	4	5	6	8	20	25
Small 0.3 µm	126,810	141,710	123,270	183,473	142,485	96,006	105,702	66,055
Medium 0.5 µm	15,834	23,645	16,581	20,623	15,967	26,018	11,924	9008
Large 5 µm	190	315	357	118	220	203	155	188

**Table 4:** Number of particles after placement of the ECOM air mask in the patients’ elevator.

Patients elevator	Time (days)							
Measurements (*10^3^ particles/m^3^)	T0	2	3	5	6	8	20	25
Small 0.3 µm	126,377	134,401	128,113	156,234	125,827	87,211	107,986	63,311
Medium 0.5 µm	12,781	20,425	14,376	11,284	14,857	24,819	16,336	7051
Large 5 µm	222	266	213	157	172	194	184	255

**Figure 1:**
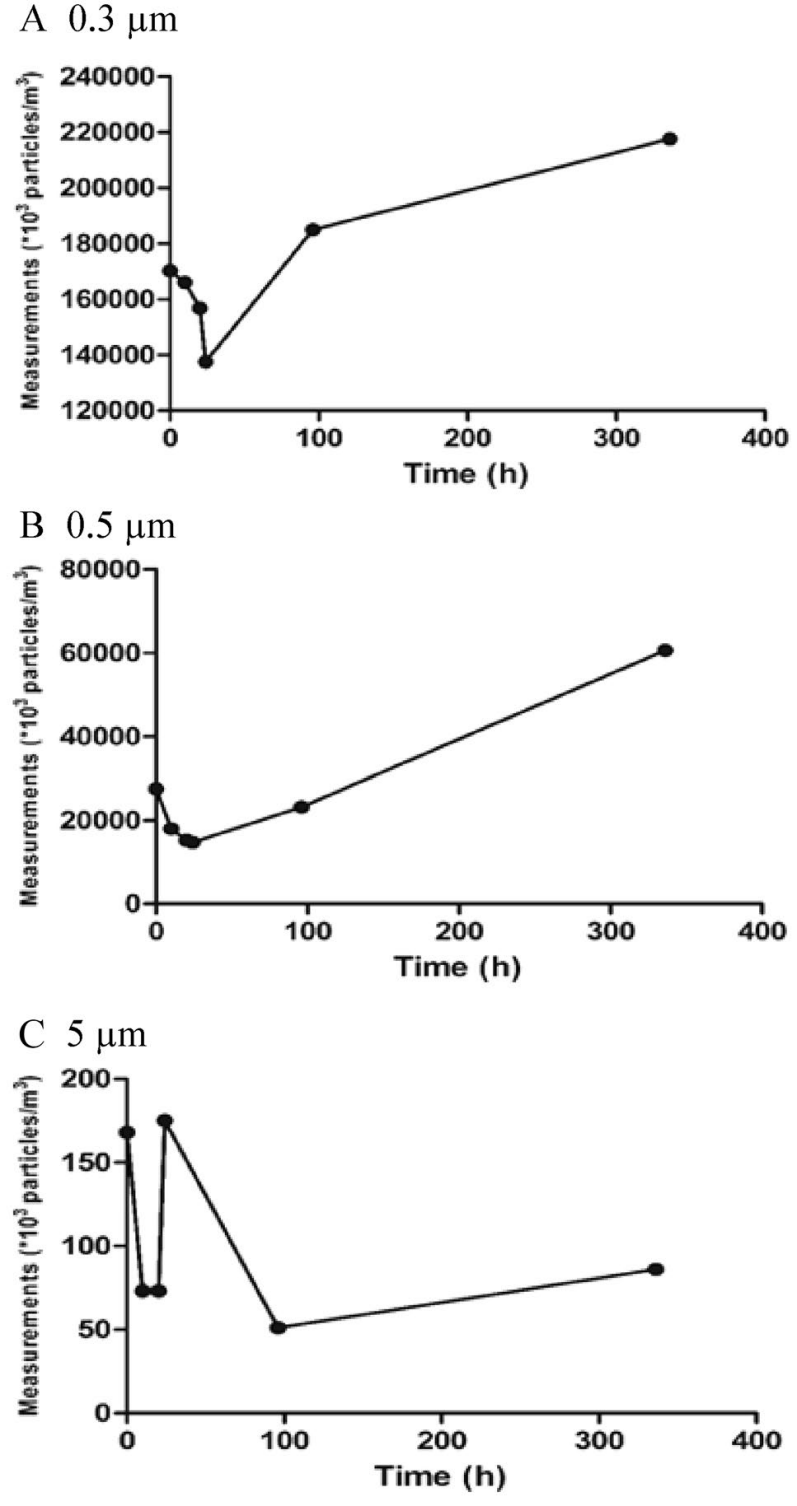
Particle count measurements after the placement of the ECOM air mask at the mobile clinic. (**A**) 0.3 µm. (**B**) 0.5 µm. (**C**) 5 µm.

In the mobile clinic, a major decrease in the particle count was detected within a few hours after mask placement and this decrease was maintained until day 4. However, this decrease was not sustained on day 15, excluding the large particle count, which was decreased by 49%, as in Table 2 and Fig. 1.

**Figure 2:**
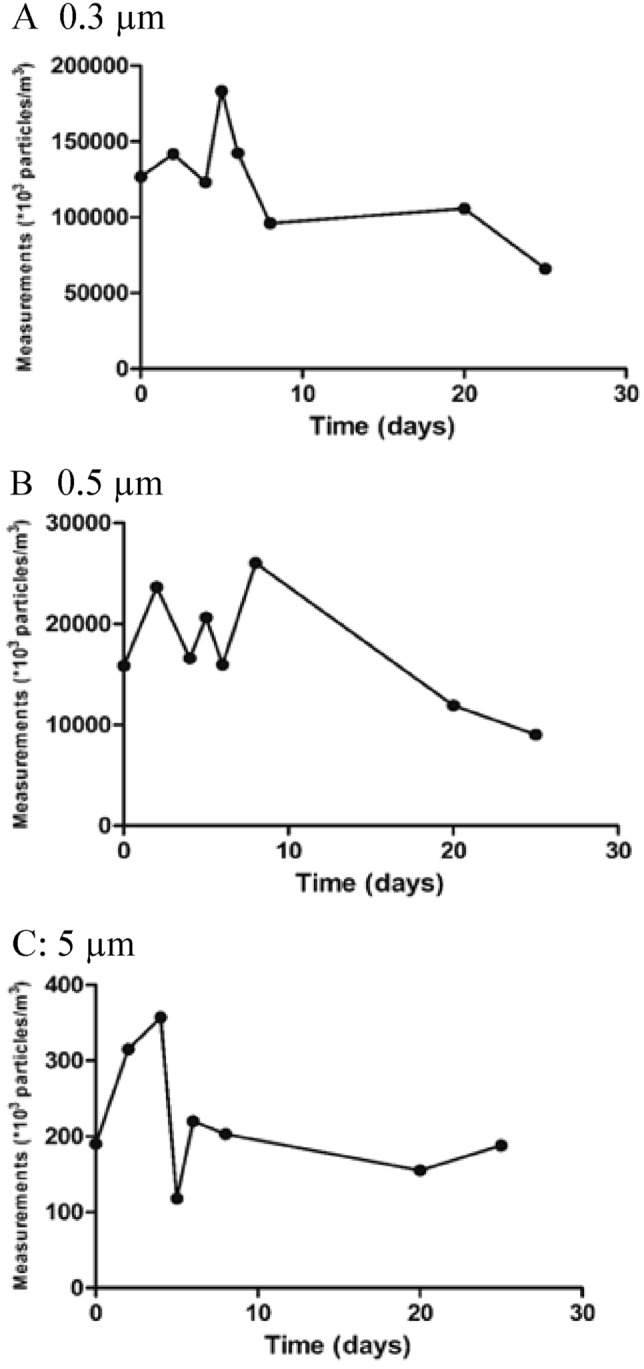
Particle count measurements after the placement of the ECOM air mask at the visitors elevator. (**A**) 0.3 µm. (**B**) 0.5 µm. (**C**) 5 µm.

In the visitors’ elevator, seen in Table 3 and Fig. 2 there were sustained decreases in the small (48%) and medium particle counts (43%), whereas the large particle count remained unchanged.

**Figure 3:**
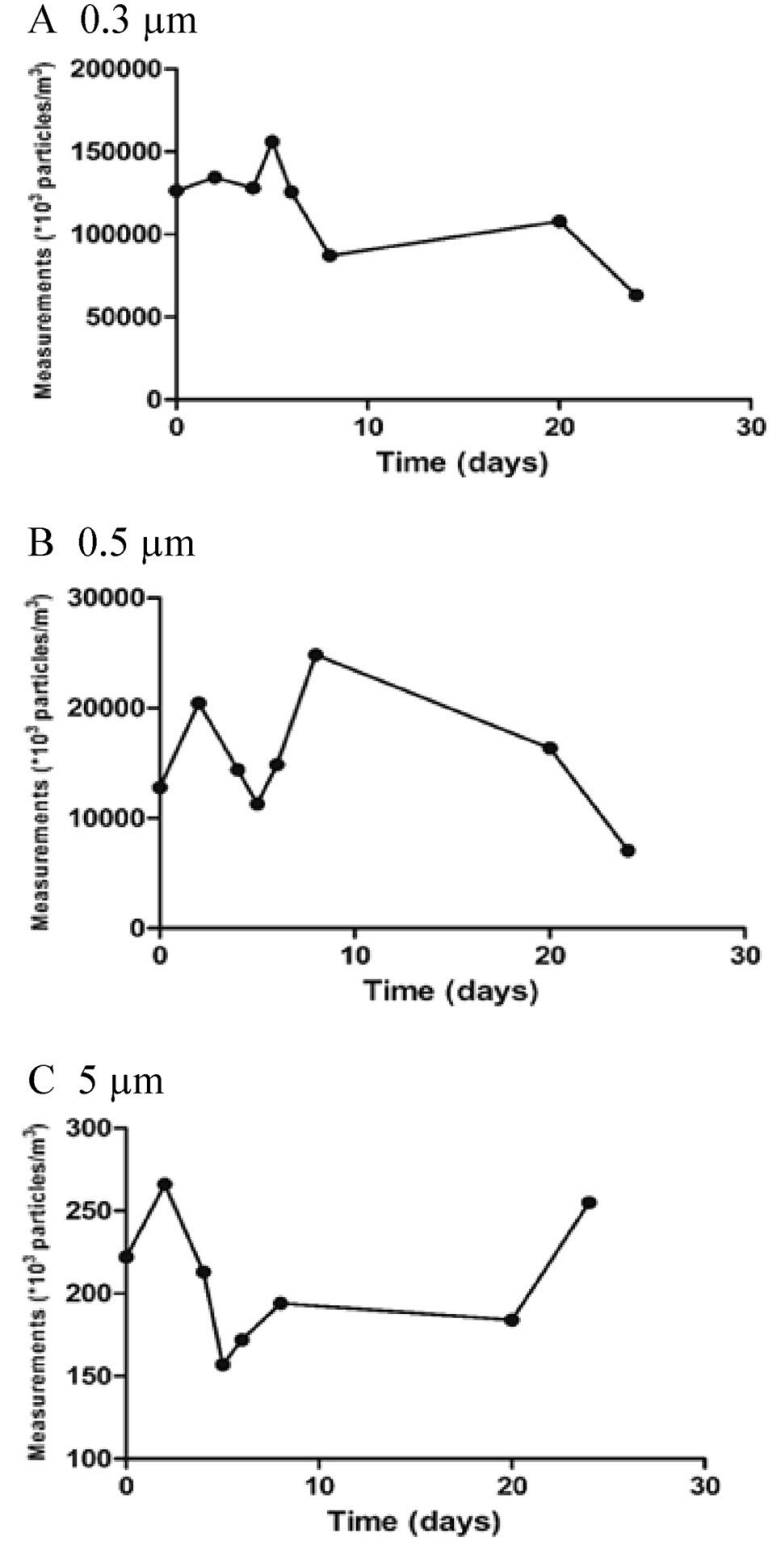
Particle count measurements after the placement of the ECOM air mask at the patients elevator. (**A**) 0.3 µm. (**B**) 0.5 µm. (**C**) 5 µm.

Similarly, in the patients’ elevator, there were sustained decreases in the small (50%) and medium particle counts (45%), whereas the large particle count remained unchanged as shown in Table 4 and Fig. 3.

## Discussion

Our experiment demonstrated the efficacy of the ECOM air mask in killing microbial pathogens at three locations and decreasing the particle load in air.

The antimicrobial properties of ClO_2_ were confirmed in this study. According to one study, these effects are attributable to its ability to denature proteins^3^. In fact, another study demonstrated that ClO_2_ is a size-selective antimicrobial agent. It can kill micro-sized organisms quickly, but it is incapable of causing measurable damage to much larger organisms because of its inability to penetrate deeply into living tissue. This might render ClO_2_ relatively safe for human use as an antiseptic^10^. ClO_2_ shares an antiseptic mechanism with natural antiseptics such as hypochlorous and hypoiodous acids. Neutrophil granulocytes use these hypohalous acids to kill bacteria after phagocytosis. ClO_2_ and hypohalous acids target and attack sulfhydryl groups, which are important for the life processes of all living systems. This explains the inability of bacteria to develop resistance against ClO_2_^11^. In addition, one study demonstrated the antibacterial effect of ClO_2_ on MDR bacteria^12^.

The antiviral effect of the gaseous form of ClO_2_ against RNA and DNA viruses has been reported^13^. Some studies suggested that this virucidal effect is attributable to the denaturing effect of ClO_2_ on amino acids such as cysteine, tyrosine and tryptophan in viral protein capsids^14^. However, because ClO_2_ exerted a virucidal effect on non-enveloped viruses, it could have other mechanisms of action that require further investigation^13^. The gaseous form of ClO_2_ also has antifungal properties and it inactivates mould and bacterial colonies^15^. Its sporicidal effect is attributable to its ability to affect cell membrane integrity and inhibit germination^16^.

Seven days after starting the experiment in this study, decreased colony counts were detected on all agar plates for samples obtained from the patients’ elevator and visitors’ elevator. Regarding mobile clinic samples, a decreased colony count was detected on LB and TSA agar plates, whereas small counts were sustained on Sab and MAC agar plates, which might be attributable to contamination.

Regarding the particle count measurement, a decrease of up to 50% was documented for most of the air samples collected. The increased particle count in the mobile clinic can be explained by the fact that the exposure of this area to outdoor settings is significant, whereas in indoor areas such as elevators, the particle count was considerably lower.

The utility of the gaseous form of ClO_2_ as an antiseptic in the hospital setting appears promising. The presence of ClO_2_ in the air reduces the number of pathogens present. The use of the ECOM air mask in the hospital setting can disinfect the air, and consequently ensure better hygiene and infection control. Our study demonstrated its efficacy as disinfectant in real life setting. Further studies are required to investigate the antiviral, antifungal and sporicidal effects of ClO_2_ in the hospital setting.

In the era of artificial intelligence, technology can complement the use of chlorine dioxide in hospital setting by developing predictive models, ensuring real time monitoring and control, and performing automated data analysis.

## Limitation

The first limitation in that our study was conducted only in one center and in specific limited sites. Multicenter studies should be performed in the future in different sites to elaborate and establish guideline for the use of such modalities.

Another limitation is that all the study sites were open areas which might have affected the particle count and culture. However, this reflects the real life setting.

## Conclusion

This study highlighted the efficacy of the ECOM air mask in decreasing particle and microbial counts at different hospital sites. The novelty of this study brings an innovative way of use of the gaseous form of ClO_2_, shown to be an effective antiseptic in hospital setting. However, further studies are needed with more data to support these results and optimize its use.

## Data Availability

All data generated or analyzed during this study are included in this published article.
