# Author Correction: Antimicrobial effects of chlorine dioxide in a hospital setting

**DOI:** 10.1038/s41598-024-53034-y

**Published:** 2024-01-30

**Authors:** Mariana Helou, Ahmad Mahdi, Antoine Abou Fayad, Ahmad Sleiman, Ghassan M. Matar, Sanaa Zoghbi, Tarek Madani, Rola Husni

**Affiliations:** 1 Division of Emergency, Department of Internal Medicine, School of Medicine, Lebanese American University, Beirut, Lebanon; 2 Division of Infectious Diseases, Department of Internal Medicine, School of Medicine, Lebanese American University, Beirut, Lebanon; 3 Center for Infectious Diseases Research, American University of Beirut, Beirut, Lebanon; 4 Infection Control Program, Lebanese American University Medical Center, Beirut, Lebanon; 5 Lebanese American University-Rizk Hospital, Beirut, Lebanon

Correction to: *Scientific Reports* 10.1038/s41598-023-49997-z, published online 18 December 2023

The original version of this Article contained an error in the name of author Ahmad Sleiman, which was incorrectly given as Sleiman Ahmad.

The original Article has been corrected.

